# Supplementary material for: Relationships between Weight Perceptions and Suicidal Behaviors in Chinese Adolescents: Results from an Ongoing School-Based Survey in Zhejiang Province
Source: Behav Sci (Basel). 2022 Dec 22;13(1):8. doi: 10.3390/bs13010008 (PMC9854802; doi:10.3390/bs13010008)
Supplement: Supplementary file 1 [file behavsci-13-00008-s001.zip › behavsci-1946365-supplementary.pdf]

Supplementary Table S1. Adjusted odds ratios (95% CIs) of suicidal ideation, plans, and attempts by the self-perceived weight status within subgroups.

	Suicidal ideation			Suicidal plans			Suicidal attempts		
	About right	Underweight	Overweight	About right	Underweight	Overweight	About right	Underweight	Overweight
Age groups (years)									
≤13	1.00	1.07(0.72-1.61)	1.39(0.98-1.97)	1.00	1.15(0.64-2.07)	1.86(1.10-3.16)*	1.00	1.01(0.49-2.09)	1.18(0.61-2.29)
14-15	1.00	1.03(0.82-1.29)	1.35(1.11-1.64)*	1.00	0.90(0.64-1.25)	1.20(0.91-1.57)	1.00	0.80(0.51-1.26)	1.66(1.19-2.30)*
≥16	1.00	1.16(0.96-1.40)	1.12(0.96-1.30)	1.00	1.07(0.81-1.41)	0.85(0.67-1.09)	1.00	1.13(0.77-1.65)	1.09(0.78-1.52)
Heterogeneity test: χ2 (P)	NA	0.65(0.72)	2.79(0.25)	NA	0.81(0.67)	8.32(0.02)	NA	1.32(0.52)	3.23(0.20)
Gender									
Boys	1.00	0.99(0.82-1.20)	0.94(0.77-1.16)	1.00	1.09(0.83-1.43)	1.00(0.74-1.35)	1.00	0.64(0.44-0.95)*	1.40(0.96-2.03)
Girls	1.00	1.16(0.95-1.42)	1.38(1.20-1.59)*	1.00	0.89(0.66-1.20)	1.07(0.87-1.33)	1.00	1.11(0.73-1.68)	1.40(1.07-1.82)*
Heterogeneity test: χ2 (P)	NA	1.26(0.26)	9.17(0.002)	NA	0.97(0.33)	0.13(0.72)	NA	3.62(0.06)	0.00(1.00)
Location of school									
Rural	1.00	1.13(0.94-1.34)	1.22(1.06-1.41)*	1.00	1.01(0.78-1.30)	1.05(0.85-1.30)	1.00	1.14(0.82-1.60)	1.31(0.99-1.72)
Urban	1.00	1.07(0.86-1.34)	1.22(1.02-1.46)*	1.00	1.03(0.74-1.42)	1.08(0.82-1.42)	1.00	0.80(0.51-1.26)	1.33(0.93-1.90)

Heterogeneity test: χ2 (P)	NA	0.14(0.71)	0.00(1.00)	NA	0.01(0.93)	0.03(0.87)	NA	1.52(0.22)	0.00(0.95)
Current smoking									
No	1.00	1.15(1.00-1.32)	1.24(1.10-1.39)*	1.00	1.08(0.87-1.33)	1.10(0.92-1.31)	1.00	1.03(0.77-1.38)	1.30(1.03-1.64)*
Yes	1.00	0.62(0.38-1.01)	0.95(0.60-1.52)	1.00	0.61(0.34-1.11)	0.65(0.36-1.18)	1.00	0.79(0.41-1.53)	1.39(0.73-2.63)
Heterogeneity test: χ2 (P)	NA	5.68(0.02)	1.19(0.28)	NA	3.17(0.08)	2.77(0.10)	NA	0.52(0.47)	0.04(0.85)
Current alcohol drinking									
No	1.00	1.12(0.95-1.33)	1.32(1.16-1.52)*	1.00	0.95(0.74-1.22)	1.15(0.94-1.43)	1.00	1.03(0.72-1.49)	1.42(1.06-1.89)*
Yes	1.00	1.04(0.81-1.33)	0.99(0.80-1.22)	1.00	1.09(0.79-1.51)	0.88(0.66-1.18)	1.00	0.99(0.67-1.47)	1.17(0.84-1.63)
Heterogeneity test: χ2 (P)	NA	0.23(0.63)	5.06(0.02)	NA	0.43(0.51)	2.14(0.14)	NA	0.02(0.89)	0.74(0.39)
Body mass index									
Underweight	1.00	1.04(0.84-1.28)	1.27(0.88-1.83)	1.00	0.78(0.57-1.05)	0.88(0.52-1.50)	1.00	0.78(0.52-1.17)	1.45(0.78-2.69)
Normal weight	1.00	1.11(0.91-1.34)	1.21(1.07-1.37)*	1.00	1.18(0.90-1.55)	1.11(0.92-1.34)	1.00	1.18(0.81-1.71)	1.33(1.04-1.69)*
Overweight / obesity	1.00	1.34(0.57-3.12)	1.14(0.63-2.09)	1.00	1.06(0.32-3.56)	1.04(0.44-2.43)	1.00	1.28(0.30-5.47)	0.97(0.35-2.67)
Heterogeneity test: χ2 (P)	NA	0.45(0.80)	0.10(0.95)	NA	3.95(0.14)	0.66(0.72)	NA	2.30(0.32)	0.44(0.80)

All models were adjusted for age, gender, location of school, school type, school performance, paternal education, maternal education, smoking, alcohol drinking, physical activity, physical measurement of body mass index, feelings of loneliness, sleep loss due to worry and experience of sadness / despair, weight control related behaviors of

exercising, dieting, taking laxatives, taking diet pills, and fasting.

* Significant results.

Supplementary Table S2. Adjusted odds ratios (95% CIs) of suicidal ideation, plans, and attempts by the accuracy of weight perceptions within subgroups.

	Suicidal ideation			Suicidal plans			Suicidal attempts		
	Accurate	Underestimated	Overestimated	Accurate	Underestimated	Overestimated	Accurate	Underestimated	Overestimated
Age groups (years)									
≤13	1.00	1.14(0.72-1.79)	1.34(0.96-1.86)	1.00	1.21(0.61-2.39)	1.65(1.02-2.67)*	1.00	0.90(0.35-2.27)	1.08(0.59-1.98)
14-15	1.00	1.02(0.77-1.36)	1.30(1.08-1.55)*	1.00	1.11(0.75-1.65)	1.37(1.06-1.76)*	1.00	1.11(0.65-1.90)	1.87(1.36-2.56)*
≥16	1.00	1.08(0.84-1.38)	1.04(0.90-1.20)	1.00	1.21(0.84-1.75)	0.91(0.73-1.14)	1.00	1.16(0.72-1.89)	1.06(0.78-1.43)
Heterogeneity test: $\chi2$ (P)	NA	0.19(0.91)	4.46(0.11)	NA	0.11(0.95)	8.21(0.02)	NA	0.22(0.89)	7.05(0.03)
Gender									
Boys	1.00	1.02(0.82-1.26)	0.98(0.81-1.17)	1.00	1.22(0.89-1.66)	1.10(0.85-1.43)	1.00	0.79(0.49-1.25)	1.33(0.93-1.90)
Girls	1.00	1.09(0.80-1.49)	1.26(1.10-1.43)*	1.00	1.13(0.72-1.76)	1.16(0.95-1.41)	1.00	2.04(1.27-3.29)*	1.42(1.10-1.82)*
Heterogeneity test: $\chi2$ (P)	NA	0.12(0.73)	4.76(0.03)	NA	0.08(0.78)	0.10(0.75)	NA	7.76(0.01)	0.09(0.77)
Location of school									
Rural	1.00	1.01(0.80-1.26)	1.12(0.98-1.28)	1.00	0.96(0.69-1.34)	1.01(0.83-1.23)	1.00	1.17(0.77-1.76)	1.27(0.98-1.64)
Urban	1.00	1.17(0.89-1.52)	1.19(1.00-1.40)	1.00	1.55(1.07-2.25)	1.39(1.07-1.79)*	1.00	1.11(0.64-1.93)	1.49(1.07-2.07)*
Heterogeneity test: $\chi2$ (P)	NA	0.67(0.41)	0.31(0.58)	NA	3.55(0.06)	2.00(0.16)	NA	0.02(0.88)	0.56(0.45)

Current smoking									
No	1.00	1.10(0.91-1.31)	1.14(1.03-1.27)*	1.00	1.17(0.90-1.53)	1.12(0.95-1.31)	1.00	1.13(0.79-1.62)	1.30(1.04-1.61)*
Yes	1.00	0.70(0.39-1.26)	1.14(0.75-1.75)	1.00	1.02(0.51-2.06)	1.16(0.68-1.96)	1.00	1.06(0.46-2.43)	1.64(0.93-2.88)
Heterogeneity test: χ2 (P)	NA	2.08(0.15)	0.00(1.00)	NA	0.13(0.72)	0.02(0.90)	NA	0.02(0.89)	0.56(0.45)
Current alcohol drinking									
No	1.00	1.08(0.88-1.33)	1.23(1.08-1.39)*	1.00	1.10(0.80-1.51)	1.20(0.99-1.46)	1.00	1.11(0.72-1.73)	1.41(1.07-1.85)*
Yes	1.00	0.98(0.72-1.33)	0.94(0.77-1.14)	1.00	1.21(0.82-1.80)	0.98(0.75-1.27)	1.00	1.18(0.72-1.93)	1.23(0.91-1.67)
Heterogeneity test: χ2 (P)	NA	0.27(0.61)	5.10(0.02)	NA	0.14(0.71)	1.47(0.23)	NA	0.03(0.86)	0.43(0.51)
Body mass index									
Underweight	1.00	NA	1.00(0.81-1.22)	1.00	NA	1.27(0.94-1.70)	1.00	NA	1.37(0.93-2.03)
Normal weight	1.00	1.11(0.91-1.34)	1.21(1.07-1.37)*	1.00	1.18(0.90-1.55)	1.11(0.92-1.34)	1.00	1.18(0.81-1.71)	1.33(1.04-1.69)*
Overweight / obesity	1.00	0.98(0.60-1.60)	NA	1.00	0.98(0.49-2.00)	NA	1.00	1.13(0.48-2.66)	NA
Heterogeneity test: χ2 (P)	NA	NA	NA	NA	NA	NA	NA	NA	NA

All models were adjusted for age, gender, location of school, school type, school performance, paternal education, maternal education, smoking, alcohol drinking, physical activity, physical measurement of body mass index, feelings of loneliness, sleep loss due to worry and experience of sadness / despair, weight control related behaviors of exercising, dieting, taking laxatives, taking diet pills, and fasting.

* Significant results.

Supplementary Table S3. Sensitivity analyses: Adjusted odds ratios (95% CIs) of suicidal ideation, plans, and attempts by the self-perceived weight status and accuracy of weight perceptions among adolescents without smoking and alcohol

drinking.

	Model 1	Model 2	Model 3	Model 4
Excluding adolescents with smoking and alcohol drinking				
**Suicidal ideation**				
Self-perceived weight status				
About right	1.00	1.00	1.00	1.00
Underweight	1.21 (1.05-1.39)	1.13 (0.96-1.32)	1.07 (0.91-1.26)	1.13 (0.96-1.34)
Overweight	1.54 (11.37-1.73)*	1.62 (1.42-1.84)*	1.51 (1.32-1.72)*	1.33 (1.16-1.52)*
Accuracy of weight perceptions				
Accurate	1.00	1.00	1.00	1.00
Underestimated	1.06 (0.87-1.29)	1.06 (0.87-1.30)	1.03 (0.83-1.26)	1.10 (0.89-1.36)
Overestimated	1.40 (1.25-1.56)*	1.43 (1.27-1.61)*	1.38 (1.23-1.56)*	1.23 (1.09-1.40)*
**Suicidal plans**				
Self-perceived weight status				
About right	1.00	1.00	1.00	1.00
Underweight	1.13(0.91-1.42)	1.01(0.79-1.29)	0.95 (0.73-1.22)	0.97 (0.75-1.25)

Overweight	1.32(1.09-1.60)*	1.42(1.16-1.74)*	1.28 (1.04-1.58)*	1.14 (0.92-1.41)
Accuracy of weight perceptions				
Accurate	1.00	1.00	1.00	1.00
Underestimated	1.08 (0.79-1.47)	1.12 (0.81-1.53)	1.07 (0.78-1.48)	1.09 (0.79-1.52)
Overestimated	1.34 (1.12-1.61)*	1.36 (1.13-1.64)*	1.30 (1.07-1.57)*	1.17 (0.96-1.43)
**Suicidal attempts**				
Self-perceived weight status				
About right	1.00	1.00	1.00	1.00
Underweight	1.04 (0.73-1.46)	1.03 (0.71-1.50)	1.01 (0.69-1.47)	0.96 (0.66-1.41)
Overweight	1.62 (1.24-2.12)*	1.62 (1.22-2.15)*	1.48 (1.11-1.97)*	1.38 (1.03-1.86)*
Accuracy of weight perceptions				
Accurate	1.00	1.00	1.00	1.00
Underestimated	1.19 (0.77-1.86)	1.13 (0.72-1.76)	1.12 (0.71-1.75)	1.05 (0.66-1.65)
Overestimated	1.46 (1.13-1.89)*	1.57 (1.20-2.05)*	1.48 (1.13-1.94)*	1.42 (1.07-1.88)*

Model 1 adjusted for age, gender, location of school, school type, school performance, paternal education and maternal education; model 2 adjusted for model 1 plus health behaviors of physical activity, and physical measurement of body mass index; model 3 adjusted for model 2 plus mental health-related characteristics of feelings of loneliness, sleep loss

due to worry and experience of sadness / despair; model 4 adjusted for model 3 plus weight control related behaviors of exercising, dieting, taking laxatives, taking diet pills, and fasting.

* Significant results.
